# Pedot:PSS/Graphene Oxide (GO) Ternary Nanocomposites for Electrochemical Applications

**DOI:** 10.3390/molecules28072963

**Published:** 2023-03-26

**Authors:** Giuseppe Greco, Antonella Giuri, Sonia Bagheri, Miriam Seiti, Olivier Degryse, Aurora Rizzo, Claudio Mele, Eleonora Ferraris, Carola Esposito Corcione

**Affiliations:** 1 Department of Engineering for Innovation, University of Salento, Edificio P, Campus Ecotekne, s.p. 6 Lecce-Monteroni, 73100 Lecce, Italy; 2 CNR-NANOTEC-Istituto di Nanotecnologia, Polo di Nanotecnologia, c/o Campus Ecotekne, via Monteroni, 73100 Lecce, Italy; 3 Department of Mechanical Engineering, Katholieke Universiteit Leuven, 2860 Sint-Katelijne Waver, Belgium

**Keywords:** ternary GGO-PEDOT nanocomposite, ternary AAGO-PEDOT nanocomposite, green methods, reduced graphene oxide, thin films, Aerosol Jet^®^ printing

## Abstract

Among conductive polymers, poly(3,4 ethylenedioxythiophene) polystyrene sulfonate (PEDOT:PSS) has been widely used as an electrode material for supercapacitors, solar cells, sensors, etc. Although PEDOT:PSS-based thin films have acceptable properties such as good capacitive and electrical behaviour and biocompatibility, there are still several challenges to be overcome in their use as an electrode material for supercapacitors. For this reason, the aim of this work is to fabricate and characterise ternary nanocomposites based on PEDOT:PSS and graphene oxide (GO), blended with green additives (glucose (G) or ascorbic acid (AA)), which have the benefits of being environmentally friendly, economical, and easy to use. The GO reduction process was first accurately investigated and demonstrated by UV-Vis and XRD measurements. Three-component inks have been developed, and their morphological, rheological, and surface tension properties were evaluated, showing their printability by means of Aerosol Jet^®^ Printing (AJ^®^P), an innovative direct writing technique belonging to the Additive Manufacturing (AM) for printed electronics applications. Thin films of the ternary nanocomposites were produced by drop casting and spin coating techniques, and their capacitive behaviour and chemical structures were evaluated through Cyclic Voltammetry (CV) tests and FT-IR analyses. CV tests show an increment in the specific capacitance of AAGO-PEDOT up to 31.4 F/g and excellent overtime stability compared with pristine PEDOT:PSS, suggesting that this ink can be used to fabricate supercapacitors in printed (bio)-electronics. The inks were finally printed by AJ^®^P as thin films (10 layers, 8 × 8 mm) and chemically analysed by FT-IR, demonstrating that all components of the formulation were successfully aerosolised and deposited on the substrate.

## 1. Introduction

Over the last few years, the integration of biomolecules within electronic elements to produce functional devices attracted significant attention. The research field coined the term “bioelectronic” to emphasise that the world of electronics could be combined with biology and biotechnology [1]. In this context, biocompatible polymers with electrons freely retained in their backbones and generally classified as conductive polymers (CPs) have become highly attractive [2]. The source of CPs’ electrical conductivity derives from the atoms at the backbone, which are characterised by weak *π*-bonds that allow the delocalisation and free movement of electrons, which in turn promotes the formation of electric current by moving charges [3]. This characteristic of CPs makes them a good candidate to be used for electrochemical, optical, and electrical purposes. The first discovery and investigation of several important CPs, such as polyaniline (PANI), polypyrrole (PPy), polythiophene (PT), trans-polyacetylene, poly (p-phenylene vinylene) (PPV) and poly(3,4-ethylenedioxythiophene) (PEDOT), dates back to 1977 [4]. Nowadays, most CPs are treated by a process known as “doping”, in which the neutral polymer chain can be oxidised or reduced with an oxidative/reductive substituent or by donor/acceptor radicals to become either positively or negatively charged [5].

Among the CPs, poly(3,4-ethylenedioxythiophene): poly (styrene sulfonate) (PEDOT:PSS) has significant importance. PEDOT:PSS is a polymeric mixture of two monomers; polystyrene sulfonic acid (PSS), which carries a negative charge on the deprotonated sulfonyl groups and poly(3,4-ethylenedioxythiophene) (PEDOT), which is a conjugated polymer, based on polythiophene and carries positive charges [6]. Despite the high conductivity, excellent stability, and good transparency of the PEDOT polymer in the UV-Vis field, its water-insoluble characteristic complicates the deposition process of thin and uniform films. To overcome this problem, PEDOT requires a “doping” process, usually performed by the addition of a counterion to the PEDOT backbone. The most used counterion in water-based dispersion is the water-soluble polyelectrolyte poly-(styrene sulfonic acid) (PSS) that leads to the synthesis of PEDOT:PSS in the stable dispersion form.

On the other hand, the electrical conductivity and capacitive properties of PEDOT:PSS can be further enhanced by the incorporation of carbon-based fillers, such as graphene and its derivatives: graphene oxide (GO), reduced graphene oxide (rGO), exfoliated graphite or carbon nanotubes (CNTs). Previous studies reveal that PEDOT:PSS /carbon-fillers nanocomposites can be used for a wide range of applications, especially for producing (bio)-electronic devices such as supercapacitors, solar cells and strain, pressure, temperature, humid and biosensors [7]. In this paper, our main focus is GO, a layered material produced through the oxidation of graphite by several explored methods [8]. Due to the presence of hydroxyl and epoxy functional groups on the basal planes, as well as carbonyl and carboxyl groups at the edges of the planes, GO is strongly oxygenated. Such functional groups generate a strong hydrophilic material that readily exfoliates in water and yields a stable dispersion of single-layered sheets [9]. Nevertheless, the presence of these highly oxygenated functional groups in its structure makes it practically an insulator as far as electrical conductivity is concerned. Therefore, it is necessary to reduce GO into reduced graphene oxide (rGO) to remove the oxygen-containing functional groups, restore the electrical conductivity and obtain a pure graphene structure. In other words, it is necessary to increase the carbon/oxygen ratio (C/O) up to the usual value, which in GO is between 2 and 4 in GO [10]. Thermal and chemical methods are commonly used to reduce GO, exploiting high temperatures (up to 900–1100 °C) [11,12] or toxic chemical agents (i.e., hydrazine [13,14], sodium borohydride [15,16], etc.). Recently, green approaches such as UV irradiation [17,18,19], vitamin C [20,21], or glucose [22,23,24] have been proposed as reducing agents, showing interesting results in terms of reduction efficiency. For instance, the addition of ascorbic acid (AA) as a green, reducing agent prevents environmental issues resulting from extremely toxic reducing agents. Moreover, the synergistic effect of adding GO and a reducing agent can significantly improve the capacitance and cycle life of the PEDOT:PSS materials as electrodes for supercapacitors [7]. Giuri et al. used glucose (G) as a green dispersing/reducing agent to produce G-GO-PEDOT:PSS ternary nanocomposite films by drop casting technique into a silicone mould, resulting in a flexible thin film and validating the simple processability of the method; films were followed by thermal annealing for 60 min at 140 °C on a hot plate in nitrogen (N_2_) atmosphere to reduce the GO via glucose [23]. Moreover, Dua, Vineet et al. used AA as a reducing agent for producing a vapour sensor through ink-jet printing [25], and Ding et al. used rGO-TiO_2_ nanocomposite films as photoanodes for dye-sensitised solar cells after reducing GO via AA in an easy and environmentally friendly method [26].

Furthermore, Khasim et al. proved an easy and efficient way to prepare secondary-doped PEDOT:PSS/rGO nanocomposites using Ethylene glycol for flexible supercapacitor electrode applications. In particular, the conductivity of PEDOT:PSS/rGO nanocomposites is increased by several orders of magnitude for the optimal content of rGO (10 wt%) compared to pure PEDOT:PSS, and the PEDOT:PSS:EG/rGO nanocomposites with higher flexibility and conductivity were used to assemble a symmetrical double layer supercapacitor device with improved capacitive behaviour [27].

In recent years, Alreda et al. studied the Electrochemical Properties of NiCo_2_O_4_/Reduced Graphene Oxide/PEDOT:PSS Ternary Nanocomposite for producing High-Performance materials. 

Supercapacitor Electrode [28], while Chen et al. showed the excellent performance of a flexible supercapacitor based on ternary composite rGO-MoS_2_-PEDOT:PSS, which will be a promising candidate for wearable energy storage device [29].

In this work, the use of a commercial GO, rather than waste materials (ash) [30], represents a starting point for the sensible bio-electronic application. However, without excluding the possibility of exploring in the future waste products that must guarantee appropriate properties. In addition, inks from the ternary nanocomposites are formulated, and an emerging 3D printing technique, Aerosol Jet^®^ Printing (AJ^®^P), which is a rising contactless direct write approach aimed at producing fine features on a wide range of substrates [31], is applied to analyse their printability. Developed for the printed electronics industry, this technology has been recently exploited also for bioelectronics and tissue engineering [32,33,34]. The green additives (G and AA) utilised for the GO reduction process were derived from natural and renewable sources, and the process was evaluated by UV-Vis and XRD techniques. Rheological and surface tension tests were performed on the liquid formulation to investigate ink printability in AJ^®^P. In addition, the capacitive properties of the inks were investigated by cyclic voltammetry (CV) tests in phosphate-buffered saline (PBS), which is a buffer, isotonic, and non-toxic solution (pH~7.4) commonly used in biological research. Finally, the FT-IR technique was implied to validate that the components were correctly deposited and printed on the substrate.

### State of Purpose

The main objective of this work is to fabricate ternary nanocomposites based on PEDOT:PSS and GO (carbon-filler), added with green elements (glucose or ascorbic acid), to produce supercapacitor devices. Specifically, after verifying the GO reduction process, the rheological and surface tension properties of the inks were analysed to evaluate their printability and filmability. Moreover, electrochemical and stability tests were carried out to investigate their feasibility in the production of devices as supercapacitors.

## 2. Results and Discussion

### 2.1. Graphene Oxide Characterisation

Starting from the promising results on PEDOT:PSS (increased conductivity, stability, film forming, etc.) reported in previous works [19,23,24], in this paper, GO was used as a secondary doping agent for PEDOT:PSS. Two different green additives were explored to improve the GO dispersion into the polymer matrix and to reduce the carbon-filler (GO), following a mild heat treatment at 140 °C, leading to the restoration of electronic configuration within the graphene layers. Unlikely [19,23,24], a new green, reducing agent (AA) was investigated, and the annealing treatment, used to allow solvent evaporation and activate the reduction process, was performed in air, in view of the fabrication of the nanocomposites by the Aerosol Jet^®^ Printing in air.

Figure S1 shows a complete characterisation of the GO filler. XRD patterns of GO film drop-casted onto a glass substrate (Figure S1a) show a sharp peak, centred on 2θ = 9.24° (001 reflection), characteristic of GO with an interlayer spacing of 0.96 nm [35]. For higher angles, an additional broad band is visible, indicating low-ordered structures due to the chemical functionalisation of the GO sheets [36].

Figure S1b shows the UV-Vis absorption spectrum of GO film drop-casted (20 μL) on a quartz substrate after drying on a hot plate at 60 °C for a few minutes to allow the water evaporation. The GO absorption spectrum shows the characteristic peaks reported in the literature [37]: a peak at ≈230 nm (π → π* transitions of aromatic C-C bonds) and a shoulder at 300 nm (n → π* transitions of C=O bonds).

The GO morphological characterisation carried out by SEM analysis is shown in Figure S1c at two different magnifications. As can be seen, the GO sheets are not perfectly distributed on the indium tin oxide (ITO) substrate, and stacking aggregates are visible in some areas. By using ImageJ software, it was possible to estimate the size of the sheets, which varies from a minimum value of about 500 nm to a maximum value of about 2 µm with an average size of 1.4 µm.

### 2.2. Graphene Oxide Reduction by Green Agents

**Figure 1:**
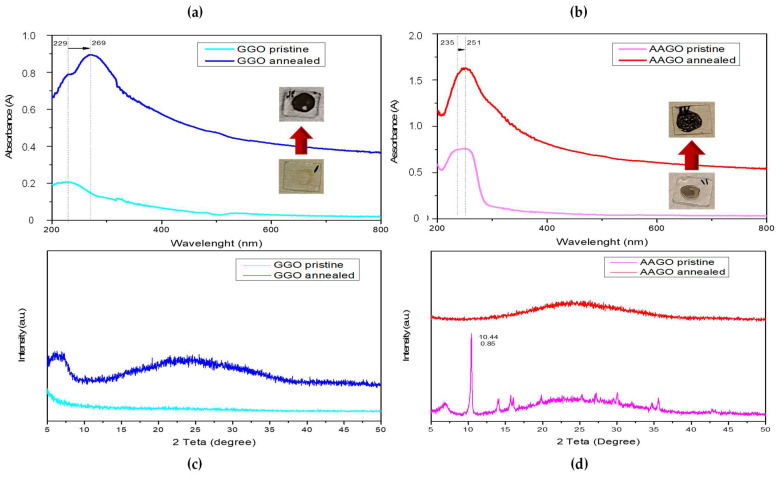
(**a**) UV-Vis absorption spectra of GGO films pre and post-thermal annealing; (**b**) UV-Vis absorption spectra of AAGO films pre and post-thermal annealing; (**c**) XRD patterns of GGO films pre and post-thermal annealing; (**d**) XRD patterns of AAGO films pre and post thermal annealing.

The effect of the selected green additives (G and AA) on GO dispersion and reduction was investigated before adding them into PEDOT:PSS, UV-Vis and XRD analyses on samples based on GGO and AAGO aqueous solutions before and after annealing thermal treatment were conducted, as reported in Figure 1.

Specifically, the GO reduction process was investigated in the presence of the green additives through UV-vis absorption tests, which consist of analysing GGO and AAGO films drop-casted onto quartz substrate by UV-Vis spectrophotometer before and after annealing at 140 °C for 1 h in air. The absorption spectra of GGO films before thermal annealing is characterised by a peak at ≈230 nm (π → π* transitions of aromatic C-C bonds) and a shoulder at 300 nm (n → π* transitions of C=O bonds) (Figure 1a). After thermal annealing, the shoulder at 300 nm (n → π* transitions of C=O bonds) disappears, and the peak at ≈230 nm (π → π* transitions of aromatic C—C bonds) shifts to 269 nm, suggesting the restoration of the electronic conjugation within the graphene sheets [24], hence the GO reduction. This phenomenon is also visibly demonstrated by the change in the GGO film’s colour from light brown to black (photographs in the inset after thermal annealing treatment which can be attributed to the absorption of K- and B-band of aromatic compounds indicating a reduction [17]).

On the other hand, the sample preparation of AAGO films before annealing showed a rapid change in the colour of the film after drying at 60 °C for a few minutes, probably due to the activation of the reduction process. Therefore, the samples were dried in air for a few hours before testing. Anyway, the AAGO pristine curve shows a large peak ranging from 235 nm to 250 nm (Figure 1b), which may be due to the beginning of the GO reduction during the mixing solution process or the drying step. The reduction in GO by AA occurs indeed at room temperature, as reported in the literature [38] and is confirmed by the slightly dark colour of the sample. After the annealing process, the absorption curve shows a well-defined peak at 251 nm, which is at a wavelength slightly lower than the value reported in the literature, which is around 260 nm. However, it demonstrates the GO reduction, as confirmed by the dark colour of the sample annealed in the inset.

To further validate the GO reduction process via green agents, an XRD analysis was carried out on GGO and AAGO films before and after thermal annealing treatment. Particularly, the diffraction XRD pattern of AAGO pristine (Figure 1d) shows a sharp peak at 10.44° (002 reflection), which is characteristic of GO shifted due to the interaction with AA [39], and a large band between 16° and 35° due to the presence of low ordered structures caused by chemical functionalisation of GO layers. Instead, the AAGO annealed pattern shows the absence of the main GO characteristic peak, suggesting that the reduction process took place correctly. So, XRD is a technique not affected by sample preparation time, such as GO-AA mixing time or air-drying time to evaluate GO reduction. In Figure 1c, the GGO pristine pattern shows the absence of any peak, as from the literature [40], and the GGO annealed pattern demonstrates that the GO reduction process took place correctly due to the disappearance of the GO characteristic peak. Moreover, there is a good intercalation as well as, presumably, the interaction of the GO layers with the additives, which can exfoliate and further distance them [24].

### 2.3. Inks Characterisation

**Figure 2:**
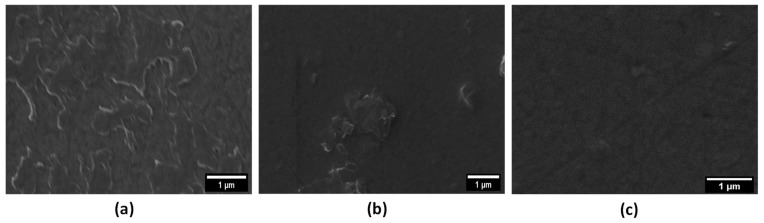
(**a**) SEM image of GO-PEDOT; (**b**) SEM image of GGO-PEDOT; (**c**) SEM image of AAGO-PEDOT.

After demonstrating the GO reduction process, the inks were analysed from a morphological point of view to investigate if the presence of green additives (glucose and ascorbic acid) facilitates the dispersion of the carbon-filler (GO) within the polymer matrix (PEDOT:PSS). SEM images of the spin-coated ink samples on ITO are shown in Figure 2.

The GO-PEDOT sample shows many micrometric aggregates (Figure 2a). Figure 2b,c clearly shows that the addition of both green additives improves the dispersion of GO sheets within the PEDOT:PSS. However, the effectiveness of the ascorbic acid, with respect to glucose, seems to be higher, as shown in Figure 2c, where very few aggregates are present. This result suggests that ascorbic acid can be considered the best dispersing agent.

**Figure 3:**
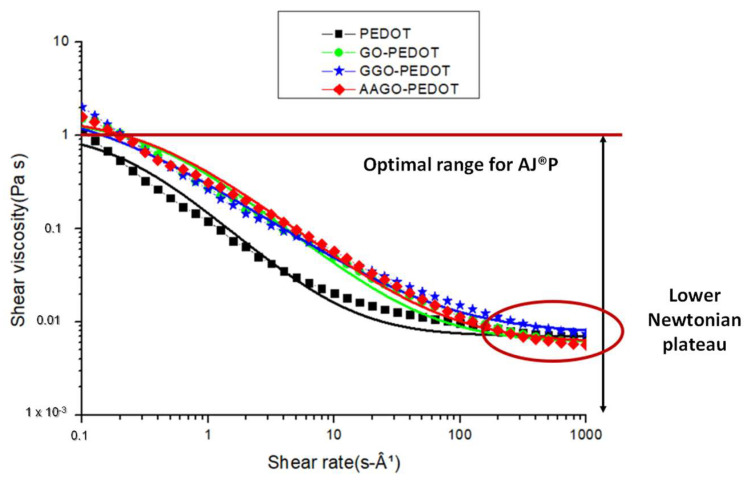
Comparison between rheological behaviour of PEDOT, GO-PEDOT, GGO-PEDOT and AAGO-PEDOT based inks and their fitting with Cross model. Moreover, the graph shows the optimum ink viscosity range for AJ^®^P and the achievement of a lower Newtonian plateau around 1000 s^−1^.

Subsequently, two characterisation tests were carried out on the developed inks with the final aim of checking their printability by AJ^®^P. Particularly, the influence of both filler (GO) and green additives (G and AA) was analysed in PEDOT:PSS-based inks in order to evaluate changes in viscosity due to their morphological structure and steric hindrance. The rheological properties of the GO-PEDOT and GO-PEDOT-additive solutions were measured at room temperature (20 °C) as a function of the shear rate. The results are shown in Figure 3. Each developed solution shows a pseudo-plastic behaviour, which is characteristic of dilute PEDOT:PSS dispersions [41], irrespectively from the presence of the dispersing agent. Particularly, the GO filler seems to induce a slight increase in viscosity of the pristine PEDOT:PSS over the entire shear rate range analysed. Indeed, at lower shear rates (0.1 s^−1^), the viscosity increases from 1.86 Pa∙s for the pristine PEDOT:PSS to 2.13 Pa∙s after adding the GO filler, instead at higher shear rates (1000 s^−1^), the addition of GO did not cause an increment of viscosity, and the two values: 0.0061 Pa∙s (PEDOT) and 0.0063 Pa∙s (GO-PEDOT) are comparable. The largest difference between the two curves, however, can be seen at intermediate shear rates, where the viscosity of pristine PEDOT:PSS tends to decrease less gradually.

The addition of G or AA to the GO-PEDOT blend has no significant influence on the viscosity; the two green additives show a similar trend, probably due to the similar molecular weight (176.12 g/mol ascorbic acid and 180.16 g/mol glucose, respectively), indeed, in both cases, at lower shear rates (0.1 s^−1^) the viscosity of GO-PEDOT decreases from 2.13 Pa∙s to 1.82 Pa∙s after adding glucose and from 2.13 Pa∙s to 1.64 Pa∙s after adding ascorbic acid. 

In contrast, at higher shear rates (1000 s^−1^), glucose leads to a slight increase in viscosity from 0.0063 Pa∙s (GO-PEDOT) to 0.0073 Pa∙s, while ascorbic acid maintains a slight decrease compared to the GO-PEDOT blend, reducing the viscosity from 0.0063 Pa∙s to 0.0056 Pa∙s. These values evidence the no-influence of additives steric hindrance on the viscosity of pristine PEDOT:PSS. 

Nevertheless, to estimate the inks viscosity values during film deposition (by spin coating at 4000 rpm), the shear rate achieved during the deposition process was calculated by using the following Equation (1):

(1) $$\dot{\gamma }=\frac{\nu }{h}$$

where *ν* is the tangential velocity (equal to the product of angular spin coating velocity *ω* and the radius of the rheometer plate), and *h* is the sample thickness (distance between the plates where the ink is placed).

The shear rate calculated, starting from Equation (1), is 13,090 s^−1^, which is out of the measurement range of the available rheometer (1000 s^−1^). Nevertheless, the curves of the ternary nanocomposites, although they have a pseudo-plastic behaviour (the viscosity decreases as the shear rate increases), show, already around 1000 s^−1^, the behaviour of a Newtonian fluid, in which the viscosity keeps constant as the shear rate increases. This result agrees with the literature [42] because polymers usually show a pseudo-plastic behaviour only in an intermediate range of shear rates, while for very low and very high values, they show a Newtonian behaviour, defined by an upper and lower Newtonian plateau, respectively, therefore, since the viscosity of both inks (GGO-PEDOT and AAGO-PEDOT) remains constant after 1000 s^−1^, we can say, with a good approximation, that the viscosity at which the inks are processed during spin coating is equal to 0.0073 Pa∙s for GGO-PEDOT ink and 0.0056 Pa∙s for AAGO-PEDOT.

To further validate the above statement, a proper theoretical model (Cross model) was applied to fit these experimental data, as reported in Figure 3. Cross [43] proposed a model to foresee the plateau values for lower and upper Newtonian viscosities, indicated as *η*_0_ and *η*_∞_, respectively:

(2) $$\eta =\eta_{\infty }+\frac{\eta_{0}-\eta_{\infty }}{1+{\left(\tau \dot{\gamma }\right)}^{m}}$$

where *τ* and *m* are model parameters and $\dot{\gamma }$ the shear rate, particularly, *τ* corresponds to the reciprocal of the shear rate at which the calculated value of *η* equals *η*_0_, and it gives an idea when the pseudo-plastic behaviour starts, while the parameter *m* is related to the power low index, *n*, by the expression: *m* = 1 − *n*. The parameter values in Equation (2), calculated by a nonlinear fitting of these experimental data shown in Figure 3, are reported in Table 1.

The values of the parameter *m*, calculated for the GO-PEDOT and GO-PEDOT additives, are close to that of pristine PEDOT. The values of *τ* for GO-PEDOT and AAGO-PEDOT inks are slightly lower than neat PEDOT, while the *τ* value of GGO-PEDOT ink is comparable with pure PEDOT, indicating approximately that the pseudo-plastic behaviour of the inks starts at similar shear rate values of PEDOT.

The inclusion of GO and additives into PEDOT:PSS does not significantly change the rheological behaviour of the pristine ink. In particular, the ink curves show viscosity values suitable for printing through AJ^®^P, as reported by Lall et al. [44]. Accordingly, the optimum viscosity range for a solution to be printed through AJ^®^P is between 0.001 and 1 Pa*s. All the inks show viscosity lower than 1 Pa*s (or very close for shear rates <0.2 s^−1^) and always higher than 0.001 Pa*s, as reported in Figure 3.

**Figure 4:**
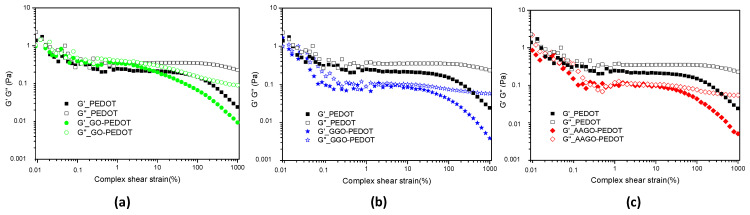
Dynamic Strain amplitude sweep tests on PEDOT compared with (**a**) GO-PEDOT, (**b**) GGO-PEDOT and (**c**) AAGO-PEDOT. Dependence of G′ (full) and G″ (empty) with the applied complex shear strain.

Referring to the dynamic tests reported in Figure 4, all the liquid formulations show a liquid-like behaviour with the loss modulus G″ that exceeds the storage modulus G′ throughout the whole shear strain range analysed, and both moduli decrease with an increase in the complex shear strain. 

In particular, after the addition of GO into PEDOT (Figure 4a), the storage (G′) and loss (G″) moduli curves show similar behaviour with comparable values. Following the addition of the green additives, the inks GGO-PEDOT (Figure 4b) and AAGO-PEDOT (Figure 4c) continue to show the same behaviour with values slightly lower but quite comparable with respect to the pristine PEDOT.

**Figure 5:**
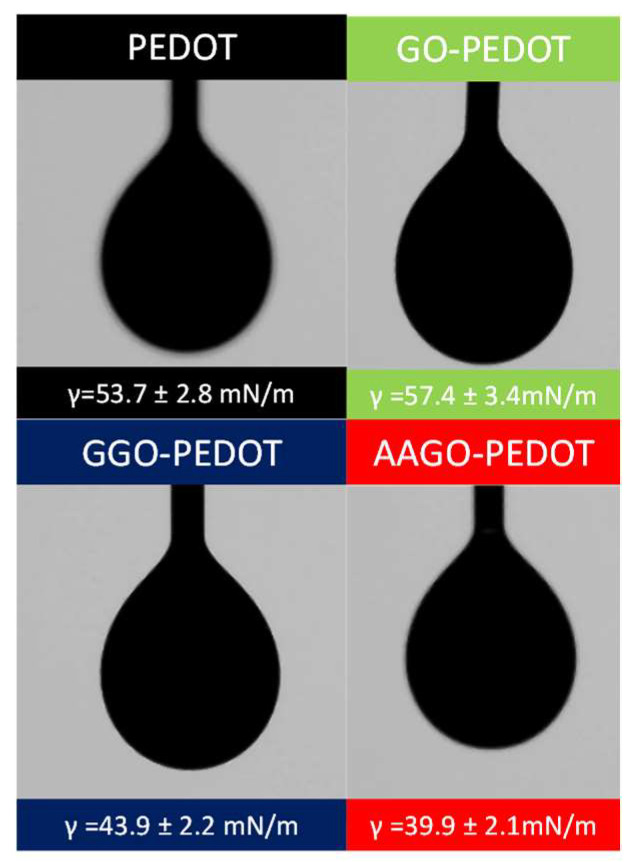
Optical pictures of surface tension measurements of pristine PEDOT, GO-PEDOT, GGO-PEDOT, AAGO-PEDOT and their droplet images.

Subsequently, the inks’ surface tension was measured to investigate if they were suitable for printing by AJ^®^P. Generally, the surface tension of a liquid determines how it changes shape under gravity, that is, how the shape of a droplet hanging off the tip of a needle changes. Figure 5 reports the surface tension values of each ink; particularly, the addition of GO in an amount of 0.05% wt/V slightly increases the surface tension of pristine PEDOT, from a value of 53.7 ± 2.8 mN/m to 57.4 ± 3.4 mN/m. On the other hand, adding the green additives results in a reduction in the inks’ surface tension; GGO-PEDOT shows a value of 43.9 ± 2.2 mN/m and AAGO-PEDOT of 39.9 ± 2.1 mN/m.

The surface tension value of GGO-PEDOT ink is slightly higher than the optimal range of surface tension that an AJ^®^P ink should have (30–40 mN/m) [45]. However, examples of inks with surface tension around 45 mN/m printed by AJ^®^P are reported in the literature [46]; moreover, the transducer in the ultrasonic configuration of our machine is capable of aerosolising even inks with higher surface tension. The AAGO-PEDOT ink shows instead a surface tension value within the optimal range required by AJ^®^P.

### 2.4. Ternary Nanocomposite Electrochemical Characterization

**Figure 6:**
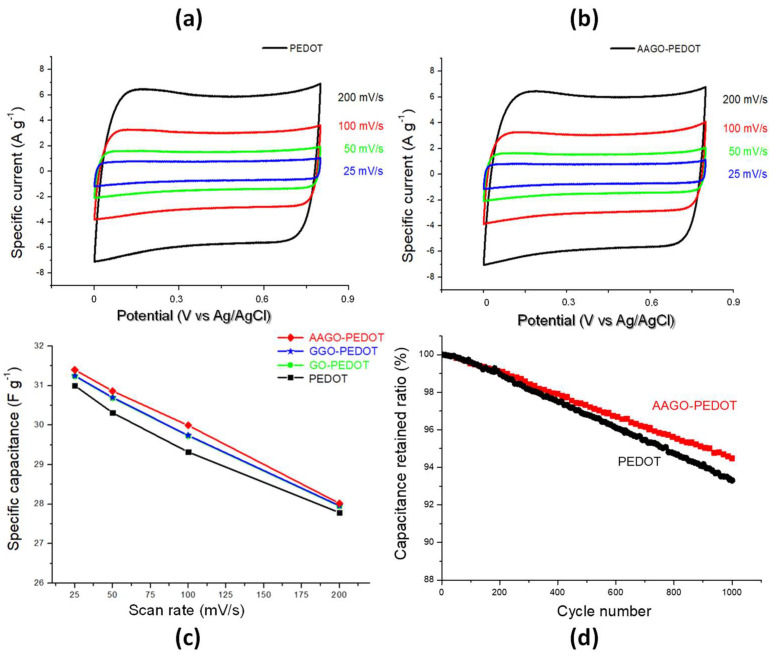
(**a**) AAGO-PEDOT cyclic voltammograms at different scan rates (25, 50, 100 and 200 mV/s). (**b**) PEDOT cyclic voltammograms at different scan rates (25, 50, 100 and 200 mV/s) (**c**) Comparison between PEDOT:PSS, GO-PEDOT, GGO-PEDOT and AAGO-PEDOT specific capacitance (**d**) Specific capacitance as a function of the cycle number for PEDOT and AAGO-PEDOT films.

The capacitive properties of the PEDOT: PSS-based inks, obtained by adding GO blended with G or AA, were studied by CV tests in PBS solution. The CV plots of PEDOT:PSS show a broad, approximately rectangular shape (Figure 6a), which can be attributed to a large double-layer capacitance [47,48]. The enclosed area of the CV curves, which corresponds to the electrode charge storage capacity, strongly depends on the ink properties [47]. The addition of GO, with or without the two green additives, apparently causes no visible difference in the obtained curves. In Figure 6b, the corresponding graph for the AAGO-PEDOT film is reported for comparison. In order to quantitatively evaluate the functional performance of the nanocomposites and then to appreciate the influence of the components of the investigated inks, the specific capacitance *C*_sp_ of the films was estimated using Equation (3) and plotted as a function of the applied CVs scan rate in Figure 6c.

(3) $$C_{sp}=\frac{\int idV}{2\;\cdot \;m\;\cdot \;\nu \;\cdot \;\Delta V}$$

In Equation (3) ∫idV is the integrated area of the CVs, ν is the scan rate, Δ*V* is the range of potential applied, and *m* is the mass of the active material.

From Figure 6c, the effect of the additives can be appreciated. The introduction of the GO in the ink results in a notable increase in *C_sp_*, as expected [49,50]. No appreciable effect is observed following the addition of glucose, whereas a slight but evident increase occurs with the addition of ascorbic acid because, as previously demonstrated, it is the best dispersing green agent. The obtained improvements could be related to a larger accessible specific surface area and increased electroactive binding sites for electrolyte ions during the charge-discharge process, giving rise to superior cyclic performance [51,52]. In particular, at lower scan rates (25 mV/s), the highest specific capacitance value is observed (31.4 F/g) for AAGO-PEDOT ink. Therefore, very good capacitive behaviour of the prepared films was demonstrated, indicating that they are potentially suitable to be used as electrode material for supercapacitors and that AAGO-PEDOT ink provides the best performance among the investigated films. Capacitive performance of the PEDOT:PSS based inks were investigated by adding GO, G and AA through CV tests in a PBS solution.

The electrochemical stability of the films was evaluated by repeating, in some representative conditions, the CV test for 1000 cycles [53,54,55]. Figure 6d shows the relative change of the specific capacitance for PEDOT and AAGO-PEDOT films as a function of the cycle number, measured from CVs obtained at 100 mV/s. A slow, gradual decrease was observed with satisfactory cyclic stability since, after 1000 cycles, 93.3% and 94.5% of the initial capacitances were retained for PEDOT and AAGO-PEDOT films, respectively. The addition of both GO and AA is therefore attractive for improving the long-term stability of PEDOT, which, although satisfactory, could be limited because of shrinkage, breaking, and cracks often appearing in conducting polymers in a sequence of many cycles [56,57]. These problems relate to volumetric changes in the polymer during the intercalation and deintercalation of counter ions [58]. From our results, the addition of a small quantity of carbon filler (GO) and green additive (AA) was proved to reduce the slow degradation in capacitance. This positive result could be due to a synergistic effect of PEDOT:PSS, GO and AA with a large accessible specific surface area and increased electroactive binding sites for electrolyte ions during the charge-discharge process, giving rise to superior cyclic performance [59].

### 2.5. Aerosol Jet^®^ Printing of Ternary Nanocomposite

After thorough characterisation, inks of the ternary nanocomposites were formulated and printed, using an emerging direct writing technology, Aerosol Jet^®^ Printing technique, in the form of thin films (about 1.5 μm of thickness).

For both ternary inks (GGO-PEDOT and AAGO-PEDOT), several thin films were produced on glass substrates (Superfrost^®^, at platen temperature 40 °C) with a square size (8 × 8 mm), serpentine infill and a number of layers equal to 10.

**Figure 7:**
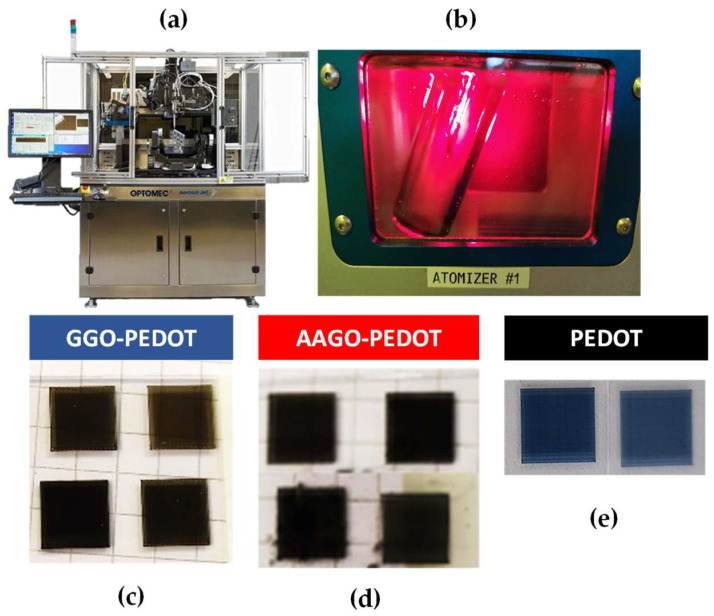
(**a**) Aerosol Jet^®^ Printing machine commercialised by Optomec Inc. (Albuquerque, NM, USA), (**b**) Atomiser in the ultrasonic configuration of Aerosol Jet^®^ Printing machine, (**c**) GGO-PEDOT thin films printed on a glass substrate, (**d**) AAGO-PEDOT thin films printed on a glass substrate, (**e**) PEDOT thin films printed on a glass substrate.

Figure 7a,b reports a representative image of the Aerosol Jet^®^ Printing system and the ultrasonic atomiser bath. The atomiser is the main component in which the aerosol formation occurs; that is, the liquid ink is first fragmented in the form of sheets or ligaments of fluid, subsequently into micro-droplets.

Figure 7c,d shows examples of the thin films produced by depositing the GGO-PEDOT and AAGO-PEDOT inks, respectively. Both inks were printed very well, particularly GGO-PEDOT shows well-defined square edges, a black colour uniformly distributed on the surface and no residual ink drops. AAGO-PEDOT thin films also show well-defined square edges and a uniform black-coloured surface, but some residual ink droplets are also visible. Standard PEDOT:PSS was printed for comparison (Figure 7e), showing uniform printed shape, and similar results have been obtained by the same authors with other PEDOT:PSS-based solutions [30,33]. However, a higher print speed equal to 25 mm/s is used for this study, which is much higher than the one used in multiple studies, that is, 1 mm/s [60,61].

Finally, in order to investigate if all of the ink’s components were able to be successfully atomised, transported, and printed onto the glass substrate during the AJ^®^P process, FT-IR analyses were performed on printed and non-printed ink powders to assess their chemical properties.

**Figure 8:**
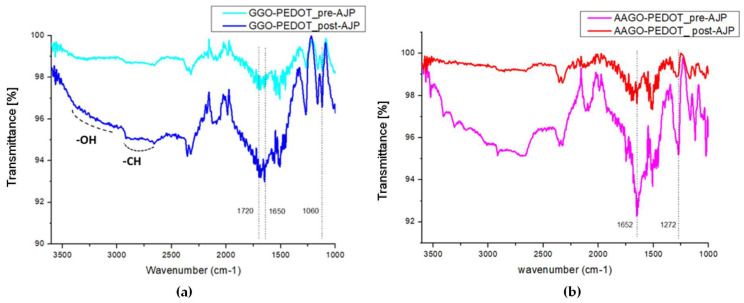
(**a**) Comparison between GGO-PEDOT pre- and post-AJ^®^P FT-IR spectrum. (**b**) Comparison between AAGO-PEDOT pre- and post-AJ^®^P FT-IR spectrum.

The FT-IR spectra of the GGO-PEDOT ternary nanocomposite pre- and post-AJ^®^P (Figure 8a), show almost the same peaks, respectively —OH stretching around 3300 cm^−1^, —CH stretching just below 3000 cm^−1^, —C=O stretching at 1650 cm^−1^ and —S=O stretching around 1160 cm^−1^. In addition, the FT-IR spectra of the AAGO-PEDOT ternary nanocomposite before and after printing by AJ^®^P show almost the same main peaks (Figure 8b). Even if in the AAGO-PEDOT post-AJ^®^P spectrum, the large band, which is typical of the stretching vibrations of the —OH groups, is less evident; the reason could be found in the evaporation of water molecules of the ink during the printing phase and/or the annealing treatment. Particularly, the FT-IR spectra of AAGO-PEDOT ternary nanocomposite highlight that the main characteristic peaks of vitamin C: ~1670 cm^−1^ (C=C double bond) and ~1320 cm^−1^ characteristics of the enol hydroxyl group (C=C bonds to —OH) stretching vibrations as reported in the literature [62] are slightly shifted towards 1652 cm^−1^ and 1272 cm^−1^ due to the interaction between all the components (PEDOT:PSS, GO and AA). Moreover, the peak shifted to 1652 cm^−1^ is very intense because it falls in the same wavelength range as the characteristic peak of GO indicating the carbonyl group (—C=O). Therefore their transmittance overlaps. However, as revealed from FT-IR spectra before and after the printing phase, all ink components successfully passed through the AJ^®^P components and were correctly deposited on the glass substrates; the result agrees with the nano nature of the additives and main components, as compared to aerosol droplets of typically 1–5 microns.

## 3. Materials and Methods

### 3.1. Materials

PEDOT:PSS was purchased by Sigma-Aldrich in water dispersion at 1.3 wt% (0.5 wt% PEDOT content, 0.8 wt% PSS content) and conductive grade. It appears as a dark blue liquid, completely miscible in water with 1 S/cm conductivity, 1000 g/cm^3^ density, 1.0–2.0 pH at 20 °C and initial melting and boiling point 0 °C and 100 °C, respectively, and as reported in its technical data sheet [63]. GO 2 mg/mL dispersion in H_2_O, used as filler, was purchased by Sigma-Aldrich, St. Louis, MO, USA; it is chloride free (purified by dialysis); it has a monolayer sheet with diameter <10 μm, 0.981 g/mL density at 25 °C and an n20/D 1.333 refractive index as reported in its technical data sheet [64]. Moreover, its characteristics include good solution processability, high hydrophilicity, low production cost and easy functionalisation because of the presence of rich active oxygen-containing functional groups. In addition, it shows a wide surface area, high chemical stability, and excellent charge carrier properties; however, it might aggregate over time to form larger particles, so it is highly recommended to sonicate if single-layer graphene oxide is required. The green additives α-D-Glucose anhydrous 96% and L-Ascorbic acid, suitable for cell culture and for plant cell culture ≥98%, were both purchased by Sigma-Aldrich. Particularly, α-D-Glucose is in powders/crystals form with 180.16 g/mol molecular weight, [α]20/D + 52°, c = 10 in H_2_O optical activity and 153–156 °C melting point as reported in its technical data sheet [65]. Instead, L-ascorbic acid is in the powder form with 176.12 g/mol molecular weight, 1.0–2.5 pH at 25 °C, 50 mg/mL water solubility, and 190–194 °C melting temperature as reported in its technical data sheet [66].

### 3.2. Ink Preparation

The ink preparation was carried out following the protocol used by Giuri et al. [23] for the preparation of ternary PEDOT:PSS-GO-G nanocomposites for flexible supercapacitors. Solutions were divided into two groups: (i) aqueous solutions and (ii) PEDOT:PSS-based solutions. The formers were prepared by adding GO (2 mg mL^−1^) into distilled water with a concentration of 0.05% (wt/vol), and the solutions were subjected to 30 min of magnetic stirring and 15 min of ultrasonication at room temperature (RT). Subsequently, 1% (wt/vol) of glucose or 0.5% (wt/vol) of ascorbic acid (vitamin C) was added to GO aqueous solutions and stirred and ultrasonicated for 15 min, respectively. The latter was prepared by adding GO (2 mg mL^−1^) into PEDOT:PSS with a concentration of 0.05% (wt/vol), and the mixtures were subjected to 90 min of magnetic stirring and 15 min of sonication in an ultrasonic bath at RT. Then, 1% (wt/vol) of glucose or 0.5% (wt/vol) Sof ascorbic acid was added to the GO-PEDOT solution, stirred and ultrasonicated for 15 min. A schematic representation of all the steps of the ink’s preparation is reported in Figure S2 of Supplementary Materials.

### 3.3. Inks Characterisation Techniques

Morphological analysis of all inks were performed on spin-coated samples of indium tin oxide (ITO) by ZEISS EVO model 40 Scanning Electron Microscopy (SEM) at EHT= 20.00 kV and high vacuum. The viscosity of all formulations was measured in a Malvern Kinexus Pro+ strain-controlled rheometer, equipped by parallel plate geometry (radius = 12.5 mm) in steady state mode with 0.4 gaps and a shear rate ranging from 0.1–1000 s^−1^ at RT. The same instrument was used to measure the viscoelastic behaviour of each ink produced. Particularly, the storage (G′) and loss (G″) moduli as a function of the shear strain (in the range of 0.01–1000%) were measured via strain amplitude sweep tests at 1 Hz of applied frequency. All the rheological tests were repeated at least three times to check the repeatability of the results. The surface tension and contact angle of the inks were measured with Ossilla Contact Angle Goniometer L2004A1, and each ink was tested at least three times.

### 3.4. Graphene Oxide Characterisation and Reduction by Green Additives

GO and GO-dispersing agents (G and AA) aqueous solutions were drop-casted on a glass substrate (2.5 × 2 cm) and analysed with a PAN analytical X’Pert-PRO diffractometer via graphite-monochromatic CuKα radiation (1.5405 Å) to determine the crystallographic structure and estimate the GO d-spacings by using the BragG′s law.

GO reduction process was evaluated by a Varian Cary 500 UV-Visible spectrophotometer; in particular GO-dispersing agent aqueous solutions were drop-casted (20 μL) onto a quartz substrate and analysed before and after thermal annealing (140 °C for 1 h), to assess the GO reduction efficiency via each green additive. Ultraviolet–visible absorption spectra were recorded in the 200–800 nm wavelength range at RT.

### 3.5. Nanocomposites Characterisation

The chemical characterisation of the samples was performed using a Compact FT-IR spectrometer Alpha II Bruker, and the spectra were recorded in the 400–4000 cm^−1^ range. FT-IR analysis was performed on the powders of each ink both before and after the printing process by AJ^®^P. 3 mL of each ink was subjected to a thermal annealing treatment at 140 °C for 1 h, then cooled down and finally cut and shredded until powders were obtained.

CV tests were employed to evaluate the capacitive properties of the materials. The electrochemical tests were carried out by a Parstat 2273 Potentiostatic/galvanostatic instrument by using the conventional three-electrode assembly. Pristine PEDOT:PSS, GO-PEDOT, AAGO-PEDOT and GGO-PEDOT inks were investigated as working electrodes. Each ink was drop-casted onto a 2 mm platinum wire surrounded by Teflon and left to be dried in the air. Meshed platinised titanium and Ag/AgCl (KCl 3M) electrodes were employed as counter and reference electrodes. All the reported potentials were vs Ag/AgCl reference electrodes. Tests were performed in 0.01M PBS at different scan rates (200, 100, 50 and 25 mV/s) in the potential range of 0V to 0.8 V. For reproducibility of results. Each test is repeated three times. Then, the electrochemical stability of the films was performed by repeating in the same representative conditions the CV test for 1000 cycles.

### 3.6. Films Fabrication

Ternary nanocomposites thin films fabrication (GGO-PEDOT and AAGO-PEDOT) was carried out using 3 different techniques: (i) Spin Coating, (ii) Drop casting and (iii) Aerosol Jet^®^ Printing. The inks produced have been deposited using a Laurell 650M Spin Coater on glass substrates (D = 12 mm), on which a plasma treatment has been previously carried out. The inks deposition phase has been repeated several times to achieve the optimal parameters to form a uniform coating on the glass substrates (3000 rpm for 60 s under vacuum). After spin coating technique, an annealing thermal treatment was performed on the samples at 140 °C for 1 h to induce GO reduction.

Drop casting films were made without the use of special tools, but only using a calibrated pipette (10–100 μL) and depositing drop by drop 108 μL of each solution on two different polymer matrix substrates. The latest technique used for film fabrication was Aerosol Jet^®^ Printing, commercialised by Optomec Inc. (Albuquerque, NM, USA). The two inks were ultrasonically atomised by using an AJ^®^P set-up at power atomisation, P = 49 V. Afterwards, pattern samples (squared shape 8 × 8 mm, with serpentine infill) were printed onto glass slides (Superfrost^®^, VWR, Leuven, Belgium) at a platen temperature 40 °C, print speed 25 mm/s, offset 3 mm, sheath gas flow 80 sccm, carrier gas flow 40 sccm, and a number of printed layers equal to 10. A post-annealing process was conducted on a hot plate (HSC Heating Magnetic stirrer Velp scientifica) at 140 °C for 1 h.

## 4. Conclusions

This paper reported the formulation and fabrication of two ternary (bio)-inks (GGO-PEDOT and AAGO-PEDOT), along with their characterisation and printability study via Aerosol Jet^®^ Printing for supercapacitor devices. The rheological characterisation and surface tension analysis of the inks showed values within the optimal range for printing thin films by AJ^®^P.

The CV tests demonstrated that AAGO-PEDOT ink has a specific capacitance higher than the pristine PEDOT:PSS, with a value of 31.4 F/g at 25 mV/s in PBS; this value is suitable for supercapacitors production for biomedical applications. Subsequently, the inks were aerosolised and printed in the form of thin square films (10 layers, 8 × 8 mm). The aerosolisation, transport, collimation and printing phases were successfully carried out for both inks, as confirmed by the FT-IR analysis performed before and after printing via AJ^®^P.

For future developments of this research work, studies should be carried out to optimise the thin films production process further, in particular the AAGO-PEDOT ones, in order to improve its printability and filmability, reducing ink waste. Moreover, other nanocomposite properties (electrical conductivity, biocompatibility) should be investigated in order to explore the feasibility of implementing these inks in (bio)-sensors and optoelectronic devices for biomedical or bio-electronic applications.

## Figures and Tables

**Table 1:** Cross model’s parameters (Equation (2)).

Sample	*η*_0_ (Pa∙s)	*η_∞_* (Pa∙s)	*τ*	*m*
PEDOT	1.1	0.007	4.78	1.24
GO-PEDOT	1.5	0.006	2.92	1.10
GGO-PEDOT	2.1	0.008	4.82	0.97
AAGO-PEDOT	1.6	0.006	3.19	1.05

## Data Availability

The data presented in this study are available on request from the corresponding authors.

## Supplementary Materials

The following supporting information can be downloaded at: https://www.mdpi.com/article/10.3390/molecules28072963/s1, Figure S1: (**a**) GO diffraction XRD patterns collected in θ-2θ scan mode drop-casted on glass, (**b**) UV-vis absorption spectrum of GO drop-casted onto quartz substrate, (**c**) SEM analysis of GO film spin-coated onto ITO substrate at two different magnifications (scale bar 1 μm and 300 nm); Figure S2: Schematic view of inks preparation process.

## Sample Availability

Samples of the nanocomposites are available from the authors.
