# Supplementary material for: Pedot:PSS/Graphene Oxide (GO) Ternary Nanocomposites for Electrochemical Applications
Source: Molecules. 2023 Mar 26;28(7):2963. doi: 10.3390/molecules28072963 (PMC10096295; doi:10.3390/molecules28072963)
Supplement: Supplementary file 1 [file molecules-28-02963-s001.zip › molecules-2254356-supplementary.pdf]

SUPPLEMENTARY INFORMATION

Pedot:PSS/Graphene Oxide (GO) Ternary Nanocomposites for Electrochemical Applications

Giuseppe Greco [1], Antonella Giuri [2], Sonia Bagheri [1], Miriam Seiti [3], Olivier Degryse [3], Aurora Rizzo [2], Claudio Mele [1], Eleonora Ferraris [3], Carola Esposito Corcione [1]*

[1] Department of Engineering for Innovation, University of Salento, Edificio P, Campus Ecotekne, s.p. 6 Lecce-Monteroni, 73100 Lecce, Italy

[2] CNR-NANOTEC-Istituto di Nanotecnologia, Polo di Nanotecnologia, c/o Campus Ecotekne, via Monteroni, I-73100 Lecce, Italy

[3] Department of Mechanical Engineering, Katholieke Universiteit Leuven, 2860 Sint-Katelijne Waver, Belgium

* Correspondence: carola.corcione@unisalento.it;

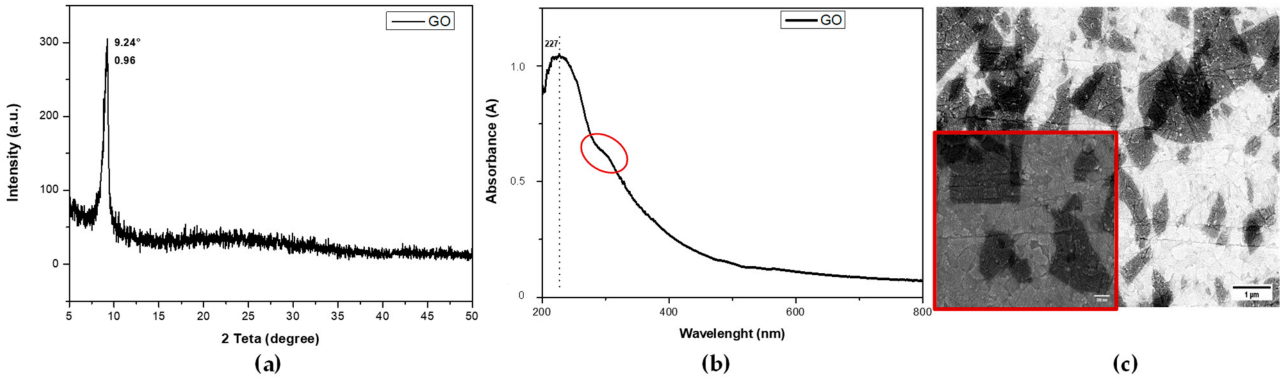

Figure S1 (a) GO diffraction XRD patterns collected in θ-2θ scan mode drop-casted on glass, (b) UV-vis absorption spectrum of GO drop-casted onto quartz substrate, (c) SEM analysis of GO film spin-coated onto ITO substrate at two different magnifications (scale bar 1 µm and 300 nm).

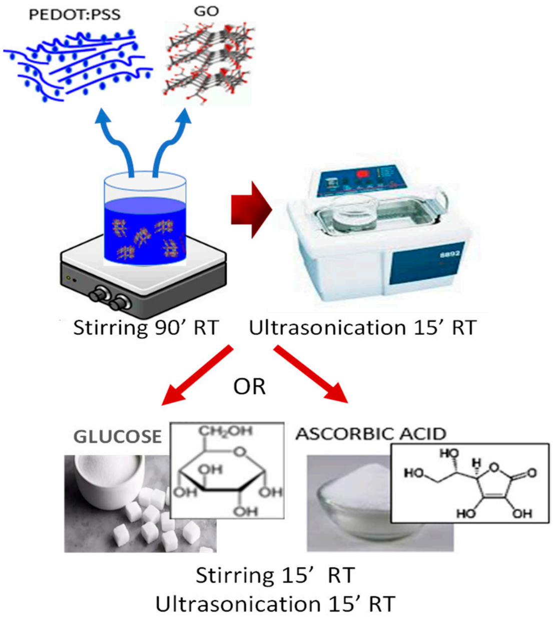

Figure S2: Schematic view of inks preparation process.
